# Fostering kappa (κ)-carrageenan hydrogels with the power of a natural crosslinker: a comparison between tender coconut water and potassium chloride (KCl) for therapeutic applications

**DOI:** 10.1007/s13205-025-04254-0

**Published:** 2025-03-14

**Authors:** Atharva Markale, Tarun Mateti, K. Likhith, S. Supriya Bhatt, K. M. Rajesh, Vishwanath Managuli, Manasa Nune, Ritu Raval, Pradeep Kumar, Goutam Thakur

**Affiliations:** 1 Department of Electrical and Electronics Engineering, Manipal Institute of Technology, Manipal Academy of Higher Education, Manipal, Udupi, Karnataka 576104 India; 2 Department of Mechanical Engineering and Materials Science, Yale School of Engineering and Applied Science, Yale University, 17 Hillhouse Avenue, New Haven, Connecticut 06520 USA; 3 Department of Bioengineering, Indian Institute of Science, C. V. Raman Road, Bangalore, Karnataka 560012 India; 4 Materials Research Centre, Indian Institute of Science, C. V. Raman Road, Bangalore, Karnataka 560012 India; 5 Department of Biomedical Engineering, Manipal Institute of Technology, Manipal Academy of Higher Education, Manipal, Udupi, Karnataka 576104 India; 6 Manipal Institute of Regenerative Medicine, Manipal Academy of Higher Education, Yelahanka, Bangalore, Karnataka 560065 India; 7 Department of Biotechnology, Manipal Institute of Technology, Manipal Academy of Higher Education, Manipal, Udupi, Karnataka 576104 India; 8 Department of Mechanical and Industrial Engineering, Manipal Institute of Technology, Manipal Academy of Higher Education, Manipal, Udupi, Karnataka 576104 India; 9 Wits Advanced Drug Delivery Platform Research Unit, Department of Pharmacy and Pharmacology, School of Therapeutic Sciences, Faculty of Health Sciences, University of the Witwatersrand, Johannesburg, 2193 South Africa; 10 Department of Pharmaceutical Sciences, Maharshi Dayanand University, Rohtak, 124001 Haryana India

**Keywords:** Polymer matrix, Polysaccharides, Biocompatibility, Antibacterial activity, Drug delivery, Tissue engineering, Diclofenac sodium

## Abstract

This study investigated the potential of tender coconut water as a natural alternative to potassium chloride (KCl) to crosslink κ-carrageenan hydrogels. κ-Carrageenan hydrogels crosslinked with tender coconut water, KCl, and their combination were formulated with diclofenac sodium as model drug, and their morphology, chemical bonding, compressive strength, water uptake capacity, degradation resistance, and cytotoxicity were assessed. The results showed that crosslinking κ-carrageenan hydrogels with both tender coconut water and KCl increased their compressive strength by up to 450%, provided excellent water retention capacity, and resulted in only 5% degradation after 20 days. Scanning electron microscopy revealed that crosslinking the hydrogel with both tender coconut water and KCl compacted its morphological structure, which remained biocompatible when tested with 3T3 cells. Infrared analysis confirmed that incorporated diclofenac sodium remained inert during preparation of the hydrogel matrices. Furthermore, the in vitro release behavior and antimicrobial properties of the hydrogels were assessed. The drug release profile from hydrogels crosslinked with both tender coconut water and KCl was sustained over 24 h. Such hydrogels also showed a unique antibacterial activity against *Staphylococcus aureus* (*S. aureus*) and *Escherichia coli* (*E. coli*)—with the activity against *E. coli* being more pronounced. In conclusion, these results confirm that crosslinking with tender coconut water and KCl is a superior alternative to just with KCl for κ-carrageenan hydrogels.

**Supplementary Information:**

The online version contains supplementary material available at 10.1007/s13205-025-04254-0.

## Introduction

Hydrogels are polymer networks with high mechanical strength employing various crosslinking techniques. Due to the presence of hydrophilic functional groups, they can imbibe large amounts of water and can be formulated using synthetic as well as natural polymers (biopolymers) (Demisli et al. 2020). One of the fascinating features of biopolymer-based hydrogels is their structural similarity with the extracellular matrix, apart from being biodegradable, biocompatible, abundant, and cost-effective (Bibire et al. 2022), (Mateti et al. 2023), making them highly suitable for tissue engineering applications (Mateti et al. 2022). In addition to their mechanical robustness, a critical aspect of these hydrogels is their ability to eliminate bacteria, for example, at the wound site to prevent infection. This can be achieved by loading them with bioactives that target essential cellular processes or structures within the bacterial cell (Mateti et al. 2021; Mitra et al. 2022).

Carrageenan is a biopolymer that is extracted from red algae (rhodophytes). Depending on the sulfate content, carrageenan is classified as λ (lambda), κ (kappa), ι (iota), ν (nu), θ (theta), and μ (mu). Of all forms, κ-carrageenan (KC) is extensively used in clinical applications due to its ability to form compact gels when its sulfate groups are crosslinked with cations, such as K^+^, Ca^2+^, and Na^+^ (Neamtu et al. 2022).

Chemical crosslinking is a versatile method used to improve the mechanical properties of biomaterial hydrogels (Panchal et al. 2022; Ramdani et al. 2023); however, chemical crosslinkers are often toxic. Instead, physical crosslinkers including electrolytes such as tender coconut water (CW) can be used as crosslinking agents. Tender coconut water has abundant potassium ions—approximately 250–300 mg of potassium per 100 g of tender coconut water (Sunil et al. 2020). Therefore, using such natural alternatives ensure sufficient crosslinking, are less likely to be cytotoxic, reduced inflammation, and is biodegradable and cost-effective.

Diclofenac sodium is a non-steroidal anti-inflammatory drug with a wide range of applications in clinical science due to its antipyretic and analgesic properties (Adeyeye and Li 1990). Although some evidence attests to its antibacterial properties (Dastidar et al. 2000), (Dutta et al. 2008a), (Dutta et al. 2004, 2007a, b, 2008b), its antimicrobial performance is yet to be fully understood and compared with a well-known antibacterial. Diclofenac sodium is also hydrophobic and has low bioavailability (Ćwiertnia 2013), and one approach to increase its availability is to stabilize it using surfactants or through incorporation in hydrogels which provide protective encapsulation to prevent precipitation or aggregation, allowing for better drug transport and controlled release and absorption (Sultana et al. 2020).

In this study, we designed κ-carrageenan hydrogels crosslinked with tender coconut water (abbreviated as KC/CW), potassium chloride (KCl; abbreviated as KC/KCl), and their combination (abbreviated as KC/CW/KCl). We analyzed their morphology, physicochemical interactions, compressive strength, water uptake capacity, degradation resistance, and cytotoxicity to assess their use as eco-friendly, natural, and cost-effective antibacterial biomaterials. The release behavior of the hydrogels was determined by loading polysorbate-80-stabilized diclofenac sodium into the hydrogels matrices, and analyzing their release at neutral pH. The antibacterial activity of the hydrogels loaded with diclofenac sodium was tested against *Staphylococcus aureus* and *Escherichia coli*, and compared to those loaded with tetracycline, an antibiotic known for its antibacterial activity against a broad range of bacteria (Rusu and Buta 2021).

## Materials and methods

### Materials

κ-Carrageenan was purchased from SNAP Natural and Alginate Products Pvt. Ltd. (Vellore, Chennai, India). KCl, polysorbate-80, and dimethyl sulfoxide were procured from Merck, India while and diclofenac sodium was purchased from Sigma Aldrich, India. Tender coconut water and castor oil were sourced from a local market in Manipal, Karnataka, India.

### Preparation of κ-carrageenan hydrogels

κ-Carrageenan hydrogels were prepared using a microemulsion method (Daniel-da-Silva et al. 2011; Rodriguez et al. 2020). Briefly, 1.5 g κ-carrageenan was added to varying amounts of polysorbate-80, and the mixture was made up to 50 ml using deionized water (Table 1). The dispersions were then stirred for 20 min at 600 rpm and 75 °C for solubilization. Castor oil was added to prevent foaming. The resulting solutions were then poured onto six-well cell culture plates (Himedia, India) and incubated in a hot air oven (Servewell Instruments Pvt. Ltd., India) for one hour at 60 °C. The plates were left at room temperature for 24 h, and the hydrogels were then removed.

**Table 1:** Chemical composition of the prepared κ-carrageenan (3%) hydrogels

Volume of polysorbate-80 (ml)	Volume of distilled water (ml)	Total volume (ml)
1	49	50
2	48	
3	47	
4	46	
5	45	
6	44	
7	43	
8	42	
9	41	
10	40	

### Preparation of diclofenac sodium-loaded crosslinked hydrogels

First, a drug solution was prepared by mixing 1 mg diclofenac sodium with 1 ml polysorbate-80 and was added to a prepared κ-carrageenan suspension as described in the method above. Crosslinker solutions comprising sterilized tender coconut water, varying molarities of KCl, and their combination were prepared (Table 2). The crosslinking solutions were added to κ-carrageenan suspensions and poured in six-well cell culture plates (Himedia, India). The obtained hydrogels were incubated in a hot air oven (Servewell Instruments Pvt. Ltd., India) for one hour at 60 °C and then stabilized at room temperature for 24 h. A control (κ-carrageenan hydrogel without crosslinking) was maintained to compare the efficacy of various crosslinkers.

**Table 2:** Chemical composition of the prepared crosslinker solutions

Molarities of KCl (M)	Volume of tender coconut water (ml)	Combination
5.0	25	25 ml 5.0 M KCl + 25 ml tender coconut water
4.5		25 ml 4.5 M KCl + 25 ml tender coconut water
4.0		25 ml 4.0 M KCl + 25 ml tender coconut water
3.5		25 ml 3.5 M KCl + 25 ml tender coconut water
3.0		25 ml 3.0 M KCl + 25 ml tender coconut water
2.5		25 ml 2.5 M KCl + 25 ml tender coconut water
2.0		25 ml 2.0 M KCl + 25 ml tender coconut water
1.5		25 ml 1.5 M KCl + 25 ml tender coconut water
1.0		25 ml 1.0 M KCl + 25 ml tender coconut water
0.5		25 ml 0.5 M KCl + 25 ml tender coconut water

### Morphological analysis

The microstructure of the hydrogels was examined using an EVO MA18 electron microscope equipped with an Oxford EDS (X-act; Zeiss, Germany) electron microscope. The samples were cut into 5 × 5 mm squares and were vacuum-dried at 40 °C (Labline, Mumbai). The microscopy was performed at a magnification of 1000 × with a 10 kV energy source after sputtering the samples with gold nanoparticles (El-Zeiny et al. 2020). A vacuum was created until the pressure reached 0.1 mbar, which was maintained by a controlled flow of argon gas. Sputtering was carried out at a current of 20 mA for 20 min. After the analysis, the samples were removed using sterile tweezers.

### Chemical interaction analysis

The Fourier-transform infrared (FTIR) spectrophotometer (Shimadzu FTIR-8400; Kyoto, Japan) was used to analyze the hydrogels' functional groups and bond linkages. The samples were dried and compressed into pellets using KBr in a ratio of 1:100 to perform the analysis (Algharib et al. 2020). The number of scans performed were 32, the resolution was set to 4 cm^−1^, and the recording range was set at 400–4000 cm^−1^.

### Mechanical strength analysis

A universal testing machine (Shimadzu EZ-SX; Kyoto, Japan) was used to assess the compressive strength of the hydrogels. Hydrogels of dimensions 35 × 15 mm were compressed at a rate of 1 mm/min under a load of 500 N (Bakarich et al. 2014). Each test group contained three samples, and the data obtained for load versus displacement were translated into nominal stress and nominal strain. Further, the stress–strain data were used to evaluate Young's modulus, compression strength, and energy. Young’s modulus and energy values were obtained by curve fitting the linear zone and by calculating the area under the curve. The compression strength corresponded to the highest nominal stress value.

### Hydrogel swelling studies

The water retention capabilities of the hydrogels were determined by weighing them at predetermined intervals after immersing them in deionized water at room temperature to calculate the extent of swelling. The hydrogels’ initial dry weight was recorded and then submerged in water for varying intervals. The swollen hydrogels were withdrawn from the water, were dabbed with blotting paper to remove any surface water, and their weights were recorded using an electronic balance. The percentage of swelling was calculated using Eq. 1 (Heidarifard et al. 2021):

1 $$\%\ {\text{Water retention}} = \frac{{w}_{t} - {w}_{0}}{{w}_{0}} \times 100$$

where *w*_*t*_ is the weight of the hydrogels immersed in water at different intervals, and *w*_0_ is the initial weight of the hydrogels.

### Hydrogel degradation behavior

A phosphate buffer solution of pH 7.4 kept at 37 °C was employed to determine the hydrogels’ degradation rate. The initial weight of the hydrogels was recorded. At predetermined intervals over 21 days, the hydrogels were removed from the phosphate buffered solution, and their weights were recorded using an electronic balance after dabbing with blotting paper (Salehi and Molzemi 2023). The percentage of degradation in the hydrogels was calculated using Eq. 2:

2 $$\%\ {\text{Degradation}} = \frac{{w}_{t} - {w}_{0}}{{w}_{t}} \times 100$$

where *w*_0_ is the initial weight of the hydrogels, and *w*_*t*_ is the weight of the hydrogels immersed in phosphate buffer solution at different time intervals.

### Cytotoxicity assessment

An MTT assay was used to determine cell viability against the hydrogels. Briefly, 3T3 cells of density 3.125 × 10^3^ cells/cm^2^ were seeded in Dulbecco’s Modified Eagle’s Medium with standard antibiotics (1% of a solution containing 10,000 UI/mL penicillin and 10 mg/mL streptomycin), supplemented with 10% fetal bovine serum, and grown on the hydrogels for 1, 3, and 5 days. The hydrogels were sterilized under ultraviolet light for 2 h before use. Thereafter, 0.5 mg/ml MTT solution was added to the sterile hydrogels, and incubated at 37 °C and 5% CO_2_ for four hours. The formazan precipitate on the hydrogels was dissolved in dimethyl sulfoxide, and the absorbance was measured at 570 nm using a multimode microplate reader (Perkin Elmer (Ensight) HH34000000). The cell viability was determined by calculating the ratio of living cells to the total cells. A control (tissue culture polystyrene (TCPS)) was maintained to compare the cytotoxicity results.

When exposed to the hydrogel environment, a live/dead viability kit was used to visualize 3T3 cell viability (Thermo Fisher, U.S.A). The materials used were disinfected using UV irradiation prior to cell seeding. The wells were incubated for one hour at 37 °C with a calcein-AM stock solution and an ethidium homodimer-1 stock solution. Calcein-AM, a cell-permeable dye, is converted to green, fluorescent calcein by live cells, while ethidium homodimer-1 binds to nucleic acids of cells with compromised membranes to create red fluorescence. A fluorescent microscope was then used to capture images (Nikon Eclipse-TE2000-U).

### Diclofenac sodium release behavior

The diclofenac sodium release behavior was analyzed using a UV–vis spectrometer (Shimadzu UV-1800; Kyoto, Japan). Diclofenac sodium-loaded hydrogels were immersed in a beaker containing 40 ml of phosphate-buffered saline (pH 7.4). The beaker was kept in a shaking incubator (Labline Instruments, India) at room temperature, and 4 ml aliquots were removed at predetermined intervals for 24 h. The sink condition was maintained by replacing 4 ml PBS to the release medium (Pan et al. 2021). The cumulative diclofenac sodium release was calculated using *Eq. *3 as described below:

3 $${\text{Cumulative diclofenac sodium release }} (\%) = \frac{{\text{Cumulative amount of diclofenac sodium released}} }{{\text{Initial amount of diclofenac sodium added}}} \times 100$$

### Antibacterial assay

The hydrogels were sterilized under ultraviolet light for two hours before further testing. Freshly cultivated *Staphylococcus aureus* (ATCC 25923) and *Escherichia coli* (ATCC 25922) were uniformly distributed over Mueller–Hinton agar, followed by placing 10 × 10 mm pieces of the hydrogels onto the agar surface. The plates were incubated for 24 h at 37 °C in a bacterial incubator, and the inhibitory zones were measured.

### Statistical analysis

All the experiments were carried out in triplicate, and the results were recorded as the arithmetic mean ± standard deviation. One-way analysis of variance (ANOVA) (GraphPad Prism 7 Software; La Jolla, USA) was used to perform the statistical analysis, and a P-value of < 0.05 was considered statistically significant.

## Results and discussion

### Selection of κ-carrageenan hydrogel and crosslinker solution composition

The sulfate groups in κ-carrageenan show an affinity for monovalent cations such as potassium and form a coil-to-helix conformational shift, followed by helix aggregation that induces the growth of crosslinked polymeric networks (Sagbas et al. 2012). The current study exploited this unique crosslinking property of κ-carrageenan to prepare hydrogels for potential biomedical applications. It was observed that when samples containing less than 6 ml polysorbate-80 were used to design κ-carrageenan hydrogels, they either remained semisolid without gelling or were difficult to remove. Alternatively, when more than 6 ml polysorbate-80 was employed, the solution was too thick to obtain an even distribution on the plate, and in some cases, gelation started to take place in the formulation container itself. Similarly, when less than 2 M KCl was used to crosslink the hydrogels, they were unstable, and when more than 2 M KCl was used, the hydrogels became excessively stiff and brittle. The same approach was also followed to determine the appropriate crosslinker combination concentration. Thus, 6 ml polysorbate-80, 2 M KCl, and 2 M KCl with tender coconut water were determined to be the best chemical compositions in their respective formulations.

### Physical and morphological properties

#### Morphology analysis

**Fig. 1:**
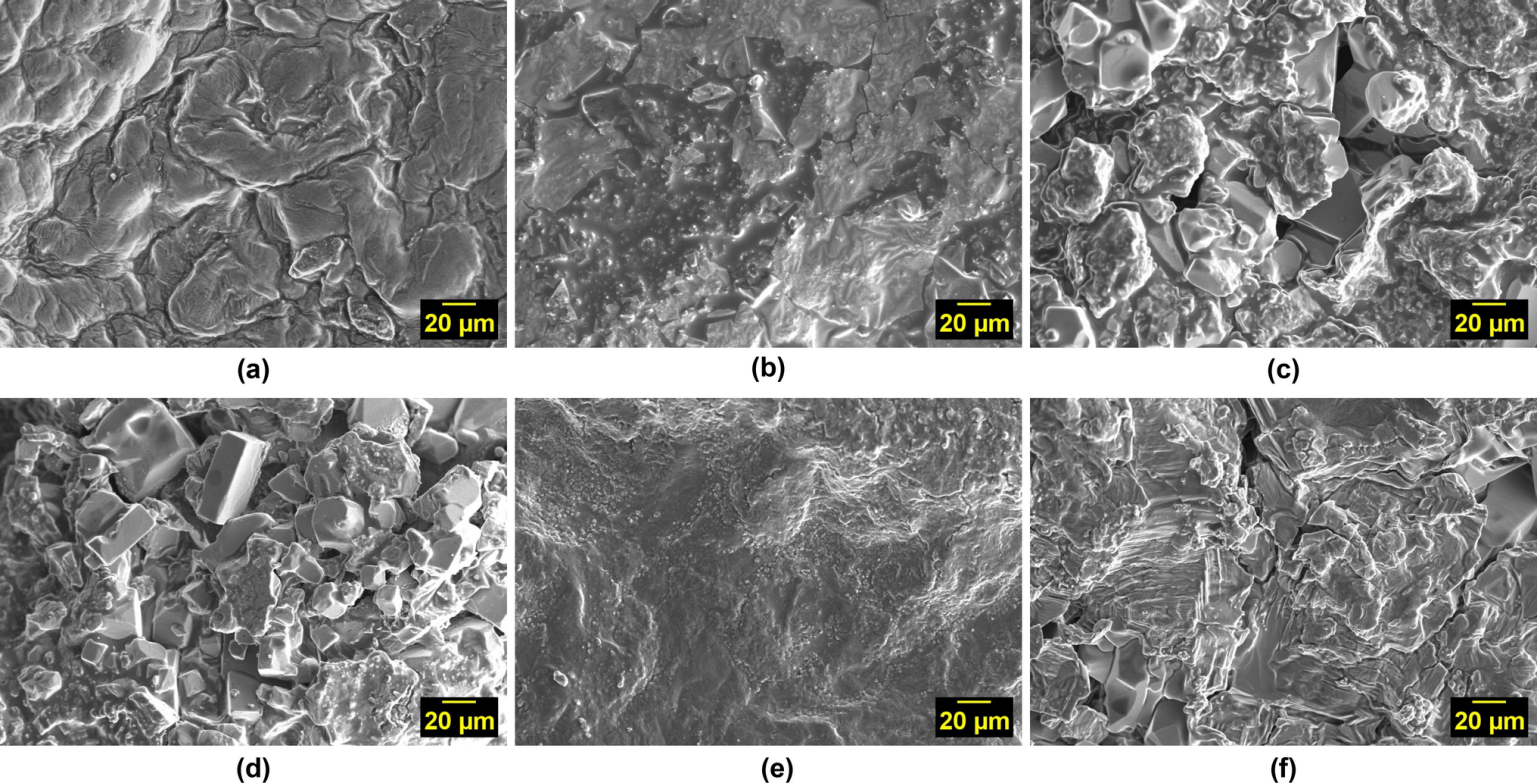
SEM micrographs of κ-carrageenan hydrogels crosslinked with **a**) tender coconut water, (**b**) tender coconut water and loaded with diclofenac sodium, (**c**) KCl, (**d**) KCl and loaded with diclofenac sodium, (**e**) tender coconut water and KCl, and (**f**) tender coconut water and KCl and loaded with diclofenac sodium

The SEM micrograph of KC/CW (Fig. 1) shows a congealed surface compared to KC hydrogels with or without diclofenac sodium (refer to online supplementary material Fig. S1), indicating that crosslinking has occurred between κ-carrageenan and tender coconut water. When diclofenac sodium was added to the hydrogels, the surface morphology became slightly rough with the compound embedded within the matrix.

The SEM micrograph of KC/KCl (Fig. 1) showed a morphology very different from those crosslinked with tender coconut water—rough, irregular, and with broad cracks. When diclofenac sodium was added to the hydrogels, the compound was trapped within the cracks of the κ-carrageenan matrix.

The SEM micrograph of KC/CW/KCl (Fig. 1) demonstrated a smooth surface; however, it developed narrow cracks wherein diclofenac sodium was trapped when the compound was added to the hydrogels. Tender coconut water appeared to have dissolved the potassium and chlorine ions, hindering the formation of crystalline structures.

It can be inferred that crosslinking κ-carrageenan with tender coconut water results in a compact morphology, whereas crosslinking with KCl results in a morphology characterized by broad cracks. The morphology of KC/CW/KCl is preferred as it forms narrow cracks that facilitate efficient diffusion from the hydrogel matrix while maintaining structural integrity. These cracks significantly increase the surface area of the hydrogel exposed to the surrounding medium, and provide additional pathways for molecules to diffuse out of the hydrogel.

#### Chemical interaction analysis

**Fig. 2:**
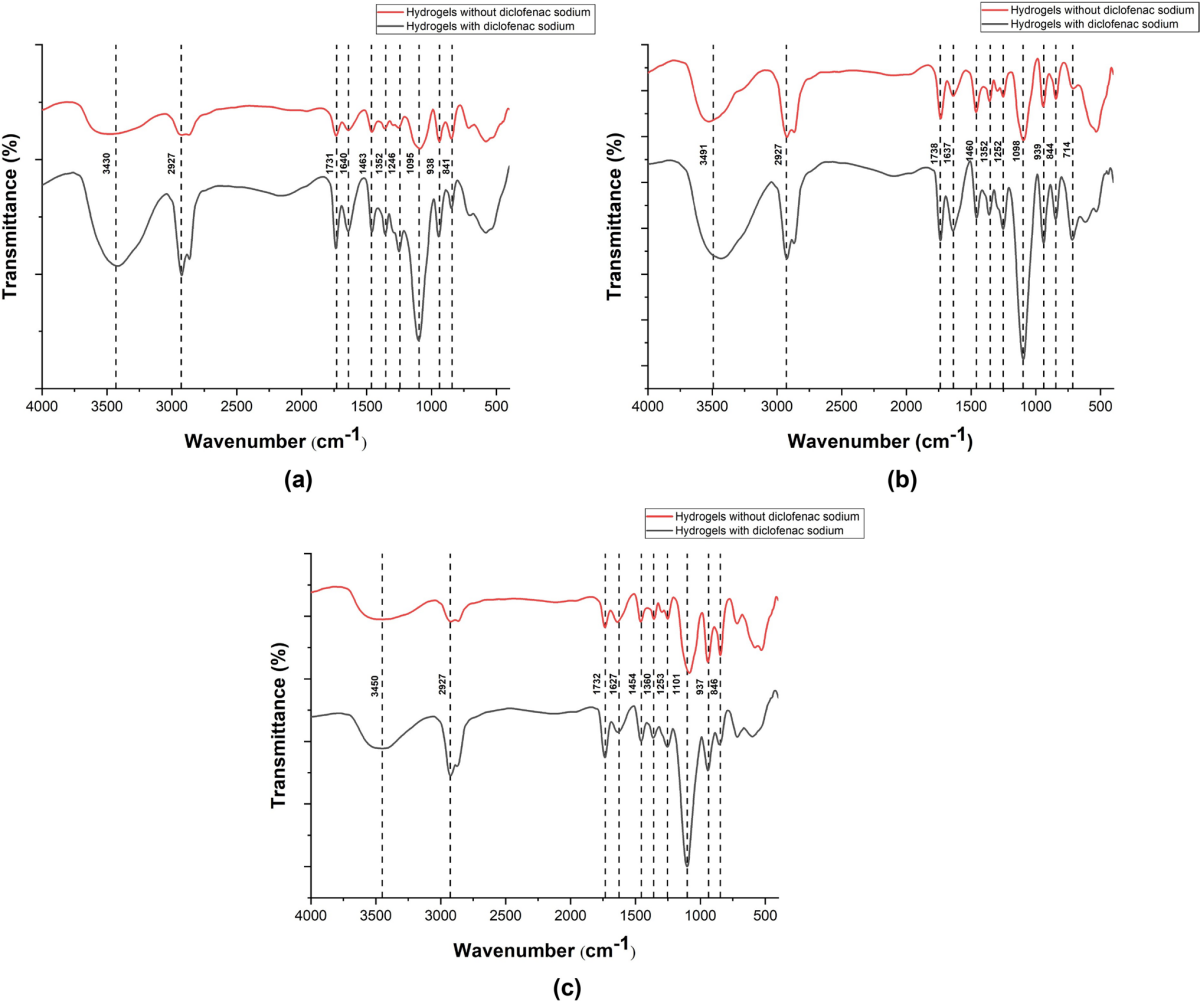
FTIR spectrum of κ-carrageenan hydrogels crosslinked using **a** tender coconut water with/without diclofenac sodium, **b** KCl with/without diclofenac sodium and **c** tender coconut water and KCl with/without diclofenac sodium

FTIR is used to study the molecular interactions between different functional groups. Figure 2 shows the FTIR spectrum of the various crosslinked hydrogels with and without diclofenac sodium. Figure S2 depicts the FTIR spectra of κ-carrageenan, polysorbate-80, and diclofenac sodium. κ-Carrageenan showed characteristic peaks at 1260 cm^−1^ and 1048 cm^−1^ that indicate S = O stretching for sulfate, 921 cm^−1^ for anhydrous glycosidic linkage, and 845 cm^−1^ for galactose-4-sulfate (Sangeetha et al. 2020). Polysorbate-80 showed C − H stretching at 2919 cm^−1^ and 2878 cm^−1^, C = O stretching at 1733 cm^−1^, C–O–C stretching at 1109 cm^−1^, and O–H stretching between 3700 and 3100 cm^−1^ (Bide et al. 2021). Diclofenac sodium demonstrated phenyl stretching between 700 and 800 cm^−1^, C − N stretching between 1000 and 1400 cm^−1^, C = C stretching at 1643 cm^−1^, C = O stretching at 1737 cm^−1^, C–H stretching between 2750 and 3000 cm^−1^, C–Cl stretching at 711 cm^−1^, and O–H stretching between 3250 and 3750 cm^−1^ (Deshmukh and Naik 2016). In Fig. 2, a few bond shifts were observed; however, new bonds were formed insignificantly. Therefore, it can be inferred that diclofenac sodium remained inert within the κ-carrageenan matrix when crosslinked with either tender coconut water, KCl, or their combination.

### Mechanical properties

#### Physicomechanical analysis

**Fig. 3:**
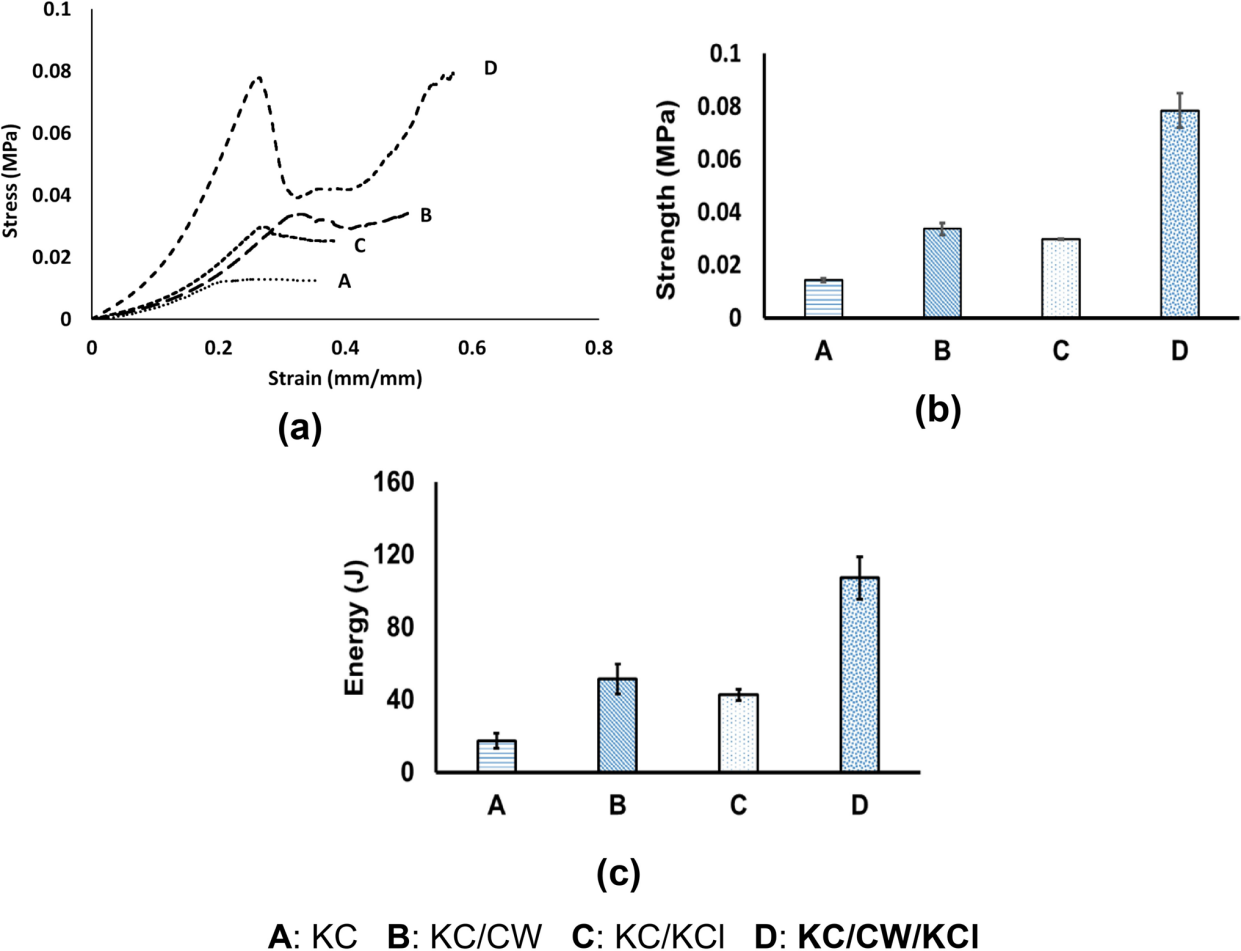
**a**, **b** Compressive behavior and strength of the uncrosslinked and crosslinked κ-carrageenan hydrogels, respectively and **c** Plot of energy absorbed by the hydrogels till the first peak in the load vs. displacement curve

Figure 3 depicts the mechanical behavior of the uncrosslinked and crosslinked κ-carrageenan hydrogels as nominal stress–strain curves, which were used to determine Young’s modulus (MPa; Table 3), compressive strength (MPa), and energy (J), characteristics that demonstrated the impact of crosslinking. The free ions and dissolved electrolytes in crosslinkers may alter the stress–strain relation, structural geometry, and types of bonds that form. From the stress–strain curve, it can be inferred that KC/CW/KCl possessed the most compressive strength, an increase of 450% compared to the KC hydrogels, and exhibited the least deformation under a high load. This was attributed to their synergistic crosslinking effect, as the combination contains high potassium ions that facilitate robust crosslinking. KC/CW exhibited the most elastic behavior compared to KC/KCl. Furthermore, tender coconut water contains more than just potassium ions with proteins, enzymes, vitamins, minerals, sugars, antioxidants, and growth factors (Dini 2019) potentially resulting in greater strength through more interconnected crosslinks than KCl alone, which primarily provides ionic crosslinking. Thus, the combination of tender coconut water and KCl promoted crosslinking to a higher degree, rendering excellent strength due to potassium ions and bioactive components forming strong bonds within the κ-carrageenan matrix.

**Table 3:** Young’s modulus of the un-crosslinked and crosslinked κ-carrageenan hydrogels

Hydrogel	Young’s modulus
KC	0.0702 ± 0.006
KC/CW	0.1032 ± 0.003
KC/KCl	0.0977 ± 0.006
KC/CW/KCl	0.3161 ± 0.018

#### Swelling studies

**Fig. 4:**
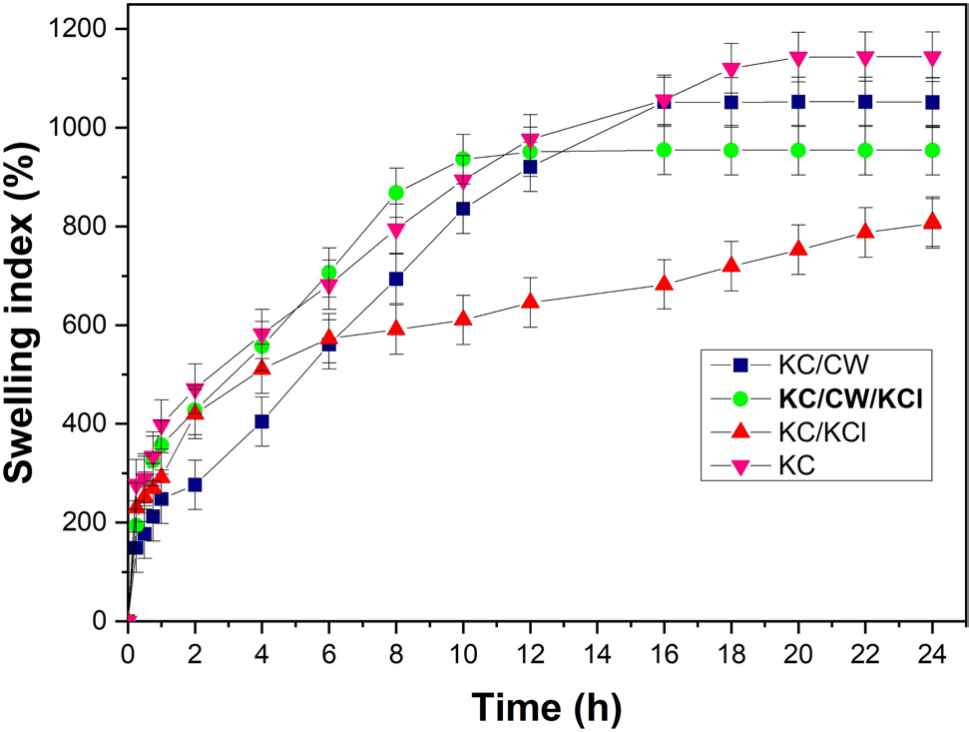
The swelling behavior of the uncrosslinked and crosslinked κ-carrageenan hydrogels

Figure 4 shows the swelling behavior of uncrosslinked and crosslinked κ-carrageenan hydrogels recorded for 24 h. At equal intervals, the swollen weights of the hydrogels were recorded, and these weights increased over time. However, after 12 h, the swelling rate decreased drastically, and the hydrogels reached saturation swelling and exhibited negligible weight change. Weight measurements were discontinued after this point, and the procedure was performed in triplicates.

The uncrosslinked and crosslinked hydrogels showed significant differences in their swelling behavior. All hydrogels displayed a controlled swelling pattern, and KC hydrogels were more porous than KC/CW, KC/KCl, and KC/CW/KCl; thus, their water uptake capacity was superior to the others. Despite possessing good water uptake properties, their low mechanical strength caused the matrix to disintegrate upon use in a saturated state. The hydrogels’ structure became rigid on crosslinking, which reduced their ability to hold water compared to uncrosslinked ones. KC/KCl showed the least swelling due to electrostatic repulsion within them that reduced their swelling capacity and dimensions (Pourjavadi et al. 2006; Mirdarikvande et al. 2014; Zhang et al. 2021). Additionally, the broad cracks in their morphology decreased their ability to draw and hold water. KC/CW displayed the most swelling among the crosslinked hydrogels; however, KC/CW/KCl were superior as they displayed excellent swelling capacity while possessing much greater strength than all other hydrogels (as discussed in “Physicomechanical analysis”). These hydrogels retained water for longer without disintegrating and maintained good moisture levels.

#### Hydrogel degradation behavior

**Fig. 5:**
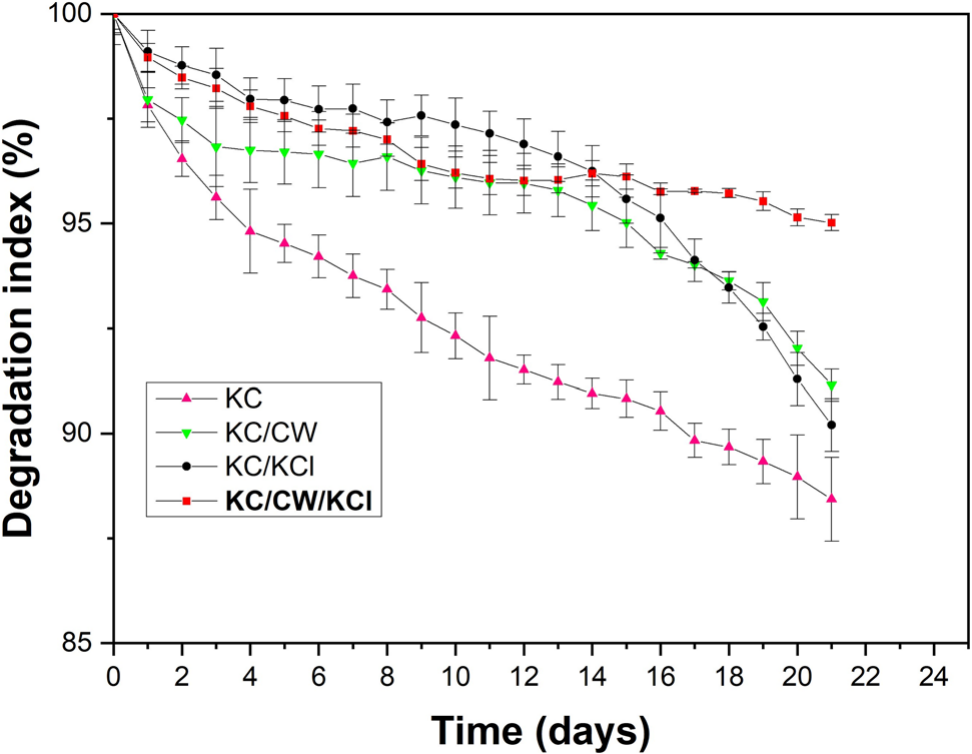
The in vitro degradation behavior of the uncrosslinked and crosslinked κ-carrageenan hydrogels at pH 7.4

Figure 5 shows the degradation behavior of the uncrosslinked and crosslinked κ-carrageenan hydrogels plotted over time at pH 7.4. KC hydrogels degraded much faster than the crosslinked hydrogels due to the absence of crosslinking, resulting in poor strength. However, KC/KCl showed a low initial degradation rate but later degraded more quickly, possibly due to the cracks on their surface expanding over time. KC/CW degraded at a rate similar to KC/KCl due to hydrolytic degradation caused by the breakdown of chemical bonds over time, primarily due to their high water uptake capacity (Kunioka and Choi 1998; Wu 2012; Pasqui et al. 2012). Surprisingly, KC/CW/KCl degraded much slower than all other hydrogels equivalent to ≈5% over 20 days. This can be attributed to their superior mechanical strength (as discussed in “Physicomechanical analysis”), and as they possessed excellent water uptake capacity (as discussed in “Swelling studies”), these can be used in applications such as wound healing to absorb exudates and omit the need for periodic replacement.

### Cytotoxicity assessment

**Fig. 6:**
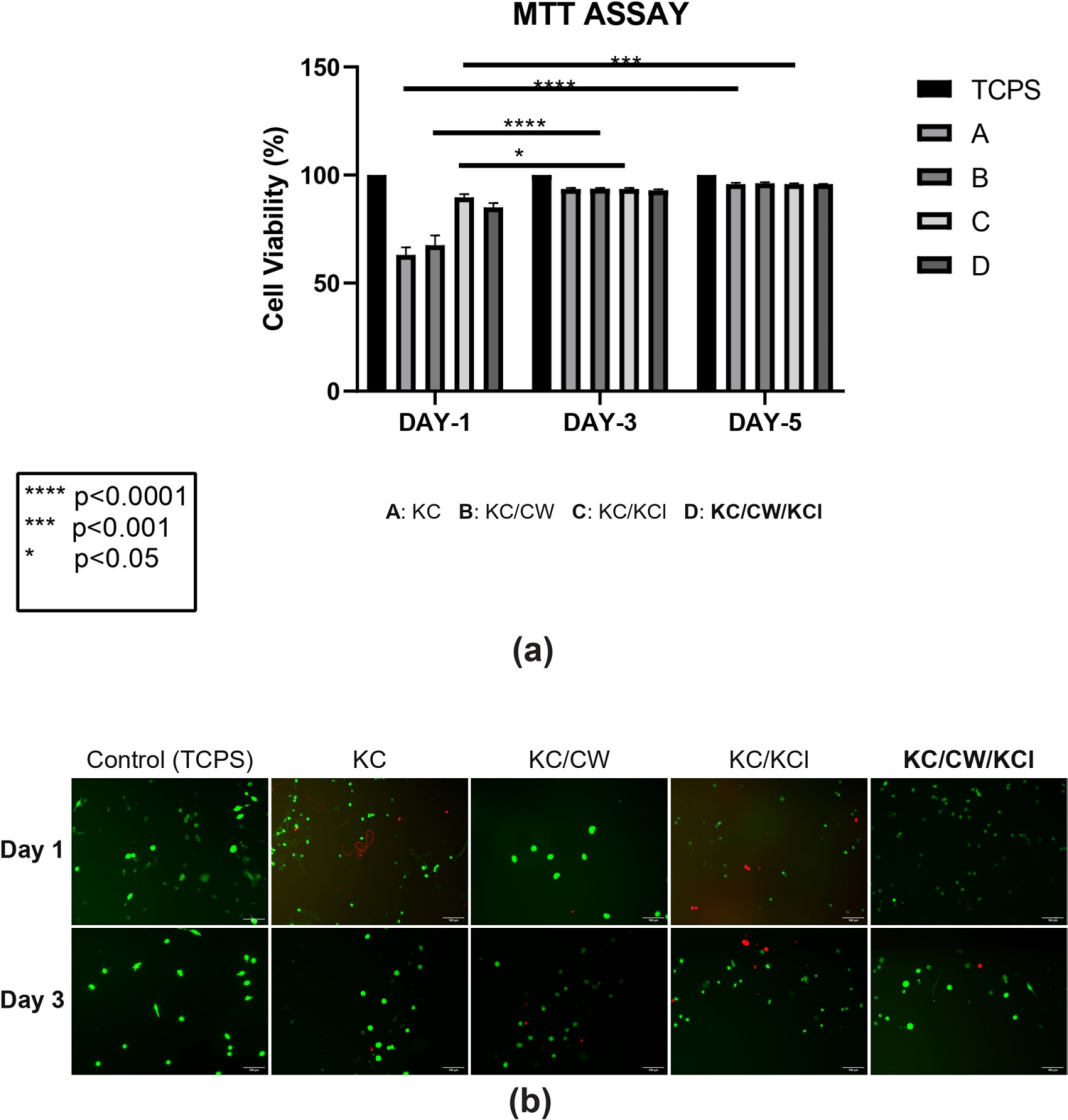
**a** Graph showing 3T3% cell viability of the uncrosslinked and crosslinked κ-carrageenan hydrogels. Each value is expressed as mean ± S.D., *n* = 3 independent experiments. **b** Live/dead images of 3T3 fibroblast cells cultured on the uncrosslinked and crosslinked κ-carrageenan hydrogels after one and three days

Figure 6 shows the cell viability of the uncrosslinked and crosslinked κ-carrageenan hydrogels after 24, 72, and 120 h, respectively. All hydrogels maintained nearly 100% cell viability on all test days, indicating insignificant cytotoxicity against 3T3 fibroblast cells. This shows that the uncrosslinked and crosslinked hydrogels are biocompatible and was further substantiated by the live/dead cell images taken one and three days after culture (Fig. 6). The cell viability results were within the acceptable limits according to ISO 10993–5–2009 (Balasubramaniam et al. 2020).

### In vitro drug release behavior

**Fig. 7:**
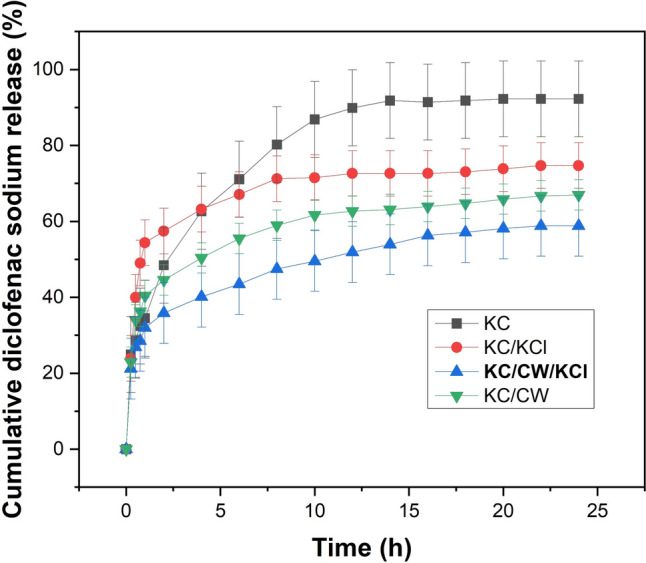
The in-vitro diclofenac sodium release profile from the uncrosslinked and crosslinked κ-carrageenan hydrogels at pH 7.4

Figure 7 shows the cumulative diclofenac sodium release from the uncrosslinked and crosslinked κ-carrageenan hydrogels plotted over time at pH 7.4. All the hydrogels displayed an initial burst release, followed by attaining a sustained release behavior over time. KC hydrogels released the most diclofenac sodium due to a drastic initial release caused by a lack of crosslinking. Out of the crosslinked hydrogels, KC/KCl released the most diclofenac sodium due to high diffusion through the broad cracks in their morphology. In contrast, KC/CW showed a sustained release behavior due to their compact structure resulting from strong crosslinking. However, KC/CW/KCl showed a desirable release behavior as the initial burst release was the least among all hydrogels, and the release behavior was steady, gradual, and sustained. Therefore, using tender coconut water and KCl reduced the undesirable burst release from κ-carrageenan hydrogels and helped maintain drug availability over a long period.

### Antibacterial assay

**Fig. 8:**
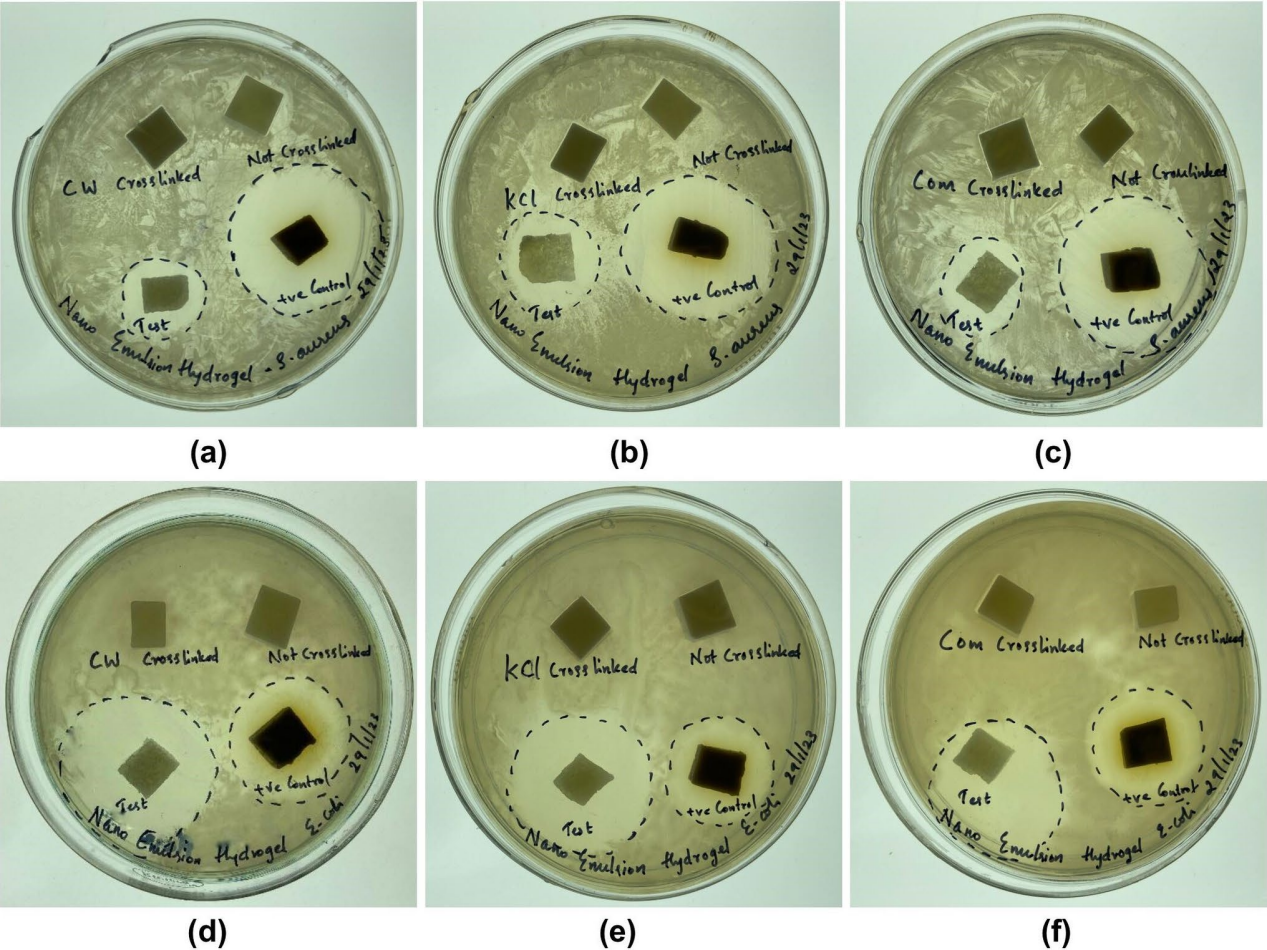
The zone of inhibition of **a**–**c** *Staphylococcus aureus* and **d**–**f** *Escherichia coli* exhibited by the uncrosslinked and crosslinked κ-carrageenan hydrogels. The ‘Test’ refers to a crosslinked hydrogel loaded with diclofenac sodium and the ‘ + ve Control’ refers to the crosslinked hydrogel loaded with tetracycline

Figure 8 shows Mueller–Hinton agar plates containing the uncrosslinked and crosslinked κ-carrageenan hydrogels with and without diclofenac sodium, a uniform spread of *Staphylococcus aureus/Escherichia coli* over the agar plates, and the hydrogel’s inhibitory zone. The ‘Test’ was a crosslinked hydrogel loaded with diclofenac sodium, whereas the ‘ + ve Control’ was a crosslinked hydrogel loaded with tetracycline.

The crosslinked hydrogels without diclofenac sodium showed an absence of an inhibition zone, indicating that the crosslinkers are inert against *Staphylococcus aureus* and *Escherichia coli*. From the ‘Test’ samples, it can be inferred that all the crosslinked hydrogels were able to release diclofenac sodium into the bacteria spread. Interestingly, diclofenac sodium was much more effective against *Escherichia coli* than *Staphylococcus aureus*, even more than tetracycline. The difference in the antibacterial effect is likely due to the bacteria's resistance to diclofenac sodium, which is believed to be due to being Gram-positive or Gram-negative.

The exact mechanism by which diclofenac sodium exerts its antibacterial effects is imprecise. Diclofenac sodium has been reported to inhibit DNA synthesis in *Escherichia coli* (Gram-negative) (Dastidar et al. 2000); however, it is unclear whether it binds directly to DNA or degrades DNA by binding to RNA polymerase. Additionally, it moderately damaged the cell membrane when tested against *Listeria monocytogenes* (Gram-positive) (Dutta et al. 2008a), and it was proposed that the compound has more than one mechanism of action that includes DNA synthesis inhibition and outer membrane damage. However, we disagree with this notion as Gram-negative bacteria possess lipopolysaccharides in their outer membrane, which act as a damage barrier to antibiotics (Zhang et al. 2013) and are absent in Gram-positive bacteria. If the damage caused to the outer membrane happened to be a mechanism of action, the antibacterial effect of diclofenac sodium would have been much more significant on *Staphylococcus aureus* than on *Escherichia coli* as it would be more permeable in the former and can damage its outer membrane more significantly. Therefore, we propose that the antibacterial activity of diclofenac sodium relies on its ability to inhibit DNA synthesis and not on its ability to disrupt the outer membrane. While our hypothesis requires further research to confirm its validity, we strongly believe in our critical analysis.

## Conclusion and prospects

This study aimed to investigate the potential of tender coconut water as a natural crosslinker alternative to KCl for κ-carrageenan by designing hydrogels crosslinked with tender coconut water, KCl, and their combination. The hydrogels were investigated for their morphology, chemical bonding, compressive strength, water uptake capacity, degradation resistance, cytotoxicity, and release behavior. The model drug chosen was diclofenac sodium, which is hydrophobic and hence was stabilized using polysorbate-80 to increase its hydrophilicity. SEM micrographs revealed that crosslinking with tender coconut water resulted in a compact morphology, whereas crosslinking with KCl led to formation of broad cracks. Notably, the crosslinker combination resulted in a morphology with narrow cracks, which promoted efficient diffusion from the hydrogel matrix. FTIR analysis confirmed that diclofenac sodium remained inert when introduced into the crosslinked hydrogel matrices, and the compressive strength analysis demonstrated that κ-carrageenan hydrogels crosslinked with both tender coconut water and KCl had enhanced strength than other hydrogels. Such hydrogels had an excellent water uptake capacity, were resistant to degradation, biocompatible, and released diclofenac sodium steadily and sustained for over 24 h. These results prove that the crosslinker combination is superior to KCl and can be used as an alternative approach to crosslink κ-carrageenan. Also, the diclofenac sodium-loaded hydrogels showed prominent antibacterial activity against *Escherichia coli,* and we suggest that diclofenac sodium exerted its antibacterial activity by relying on its ability to inhibit DNA synthesis and not on its ability to disrupt the outer membrane. However, this hypothesis may require additional evidence to validate its accuracy. It is an exciting prospect for the scientific community to explore, which would help establish the science behind our findings and persuade us to use diclofenac sodium against bacterial infections.

## Supplementary Information

Below is the link to the electronic supplementary material.

Supplementary file1 (DOCX 524 KB)

## Data Availability

The data supporting this article have been included as part of the Supplementary Information.

## References

[CR1] Adeyeye CM, Li PK (1990) Diclofenac Sodium. Analytical Profiles of Drug Substances and Excipients, Elsevier 19:123–144. 10.1016/S0099-5428(08)60366-4

[CR2] Algharib SA, Dawood A, Zhou K et al (2020) Designing, structural determination and biological effects of rifaximin loaded chitosan- carboxymethyl chitosan nanogel. Carbohydr Polym 248:116782. 10.1016/J.CARBPOL.2020.116782 32919570 10.1016/j.carbpol.2020.116782

[CR3] Bakarich SE, Balding P, Gorkin R et al (2014) Printed ionic-covalent entanglement hydrogels from carrageenan and an epoxy amine. RSC Adv 4:38088–38092. 10.1039/C4RA07109C

[CR4] Balasubramaniam MP, Murugan P, Chenthamara D et al (2020) Synthesis of chitosan-ferulic acid conjugated poly(vinyl alcohol) polymer film for an improved wound healing. Mater Today Commun 25:101510. 10.1016/J.MTCOMM.2020.101510

[CR5] Bibire T, Yilmaz O, Ghiciuc CM et al (2022) Biopolymers for surgical applications. Coatings 12:211. 10.3390/COATINGS12020211

[CR6] Bide Y, Fashapoyeh MA, Shokrollahzadeh S (2021) Structural investigation and application of Tween 80-choline chloride self-assemblies as osmotic agent for water desalination. Sci Rep 11:1–11. 10.1038/s41598-021-96199-6 34426591 10.1038/s41598-021-96199-6 PMC8382744

[CR7] Ćwiertnia B (2013) Effect of water soluble carrier on dissolution profiles of diclofenac sodium. Acta Pol Pharm 70:721–726 23923395

[CR8] Daniel-da-Silva AL, Ferreira L, Gil AM, Trindade T (2011) Synthesis and swelling behavior of temperature responsive κ-carrageenan nanogels. J Colloid Interface Sci 355:512–517. 10.1016/J.JCIS.2010.12.071 21251667 10.1016/j.jcis.2010.12.071

[CR9] Dastidar SG, Ganguly K, Chaudhuri K, Chakrabarty AN (2000) The anti-bacterial action of diclofenac shown by inhibition of DNA synthesis. Int J Antimicrob Agents 14:249–251. 10.1016/S0924-8579(99)00159-4 10773497 10.1016/s0924-8579(99)00159-4

[CR10] Demisli S, Mitsou E, Pletsa V et al (2020) Development and study of nanoemulsions and nanoemulsion-based hydrogels for the encapsulation of lipophilic compounds. Nanomaterials 10:2464. 10.3390/NANO10122464 33317080 10.3390/nano10122464 PMC7763598

[CR11] Deshmukh RK, Naik JB (2016) Optimization of spray-dried diclofenac sodium-loaded microspheres by screening design. Drying Technol 34:1593–1603. 10.1080/07373937.2016.1138121

[CR12] Dini I (2019) An Overview of Functional Beverages. In: Functional and Medicinal Beverages. Academic Press, pp 1–40

[CR13] Dutta NK, Kumar A, Mazumd K et al (2004) In vitro and in vivo antimycobacterial activity of antiinflammatory drug, diclofenac sodium. Indian J Exp Biol 3:922–927 15462188

[CR14] Dutta NK, Annadurai S, Mazumdar K et al (2007a) Potential management of resistant microbial infections with a novel non-antibiotic: the anti-inflammatory drug diclofenac sodium. Int J Antimicrob Agents 30:242–249. 10.1016/J.IJANTIMICAG.2007.04.018 17644318 10.1016/j.ijantimicag.2007.04.018

[CR15] Dutta NK, Mazumdar K, Dastidar SG, Park JH (2007b) Activity of diclofenac used alone and in combination with streptomycin against Mycobacterium tuberculosis in mice. Int J Antimicrob Agents 30:336–340. 10.1016/J.IJANTIMICAG.2007.04.016 17644321 10.1016/j.ijantimicag.2007.04.016

[CR16] Dutta NK, Mazumdar K, Baek MW et al (2008a) In vitro efficacy of diclofenac against Listeria monocytogenes. Eur J Clin Microbiol Infect Dis 27:315–319. 10.1007/S10096-007-0439-5/FIGURES/2 18188616 10.1007/s10096-007-0439-5

[CR17] Dutta NK, Mazumdar K, Seok SH, Park JH (2008b) The anti-inflammatory drug Diclofenac retains anti-listerial activity in vivo. Lett Appl Microbiol 47:106–111. 10.1111/J.1472-765X.2008.02391.X 18643914 10.1111/j.1472-765X.2008.02391.x

[CR18] El-Zeiny HM, Abukhadra MR, Sayed OM et al (2020) Insight into novel β-cyclodextrin-grafted-poly (N-vinylcaprolactam) nanogel structures as advanced carriers for 5-fluorouracil: equilibrium behavior and pharmacokinetic modeling. Colloids Surf A Physicochem Eng Asp 586:124197. 10.1016/J.COLSURFA.2019.124197

[CR19] Heidarifard M, Taghavi E, Anarjan N (2021) Preparation of nano-emulsion-based hydrogels conjugated curcumin as model functional lipid bioactive compound. J Am Oil Chem Soc 98:697–709. 10.1002/AOCS.12473

[CR20] Kunioka M, Choi HJ (1998) Hydrolytic degradation and mechanical properties of hydrogels prepared from microbial poly(amino acid)s. Polym Degrad Stab 59:33–37. 10.1016/S0141-3910(97)00181-X

[CR21] Mateti T, Likhith K, Laha A, Thakur G (2023) A critical analysis of the recent developments in multi-stimuli responsive smart hydrogels for cancer treatment. Curr Opin Biomed Eng. 10.1016/j.cobme.2022.100424

[CR22] Mateti T, Aswath S, Vatti AK et al (2021) A review on allopathic and herbal nanofibrous drug delivery vehicles for cancer treatments. Biotechnol Rep. 10.1016/j.btre.2021.e00663 10.1016/j.btre.2021.e00663 PMC8446576 34557390

[CR23] Mateti T, Laha A, Shenoy P (2022) Artificial meat industry: production methodology, challenges, and future. JOM. 10.1007/s11837-022-05316-x35228788

[CR24] Mirdarikvande S, Sadeghi H, Godarzi A et al (2014) Effect of pH, and salinity onto swelling properties of hydrogels based on H-alginate-g-poly (AMPS). Biosci Biotechnol Res Asia 11:205–209

[CR25] Mitra S, Mateti T, Ramakrishna S, Laha A (2022) A review on curcumin-loaded electrospun nanofibers and their application in modern medicine. JOM. 10.1007/s11837-022-05180-9 35228788 10.1007/s11837-022-05180-9 PMC8867693

[CR26] Neamtu B, Barbu A, Negrea MO et al (2022) Carrageenan-based compounds as wound healing materials. Int J Mol Sci 23:9117. 10.3390/IJMS23169117 36012381 10.3390/ijms23169117 PMC9409225

[CR27] Pan R, Liu G, Zeng Y et al (2021) A multi-responsive self-healing hydrogel for controlled release of curcumin. Polym Chem 12:2457–2463. 10.1039/D1PY00176K

[CR28] Panchal R, Mateti T, Likhith K et al (2022) Genipin cross-linked chitosan–PVA composite films: an investigation on the impact of cross-linking on accelerating wound healing. React Funct Polym. 10.1016/j.reactfunctpolym.2022.105339

[CR29] Pasqui D, De Cagna M, Barbucci R (2012) Polysaccharide-based hydrogels: the key role of water in affecting mechanical properties. Polymers (Basel) 4:1517–1534. 10.3390/POLYM4031517

[CR30] Pourjavadi A, Barzegar S, Mahdavinia GR (2006) MBA-crosslinked Na-Alg/CMC as a smart full-polysaccharide superabsorbent hydrogels. Carbohydr Polym 66:386–395. 10.1016/J.CARBPOL.2006.03.013

[CR31] Ramdani R, Rao AM, Pokharel M et al (2023) Curcumin-laden crosslinked chitosan–PVA films: the synergistic impact of genipin and curcumin on accelerating wound closure. JOM. 10.1007/S11837-023-06123-8/FIGURES/10

[CR32] Rodriguez S, Torres FG, Arroyo J et al (2020) Synthesis of highly stable κ/ι-hybrid carrageenan micro- and nanogels via a sonication-assisted microemulsion route. Polym Renewable Resour 11:69–82. 10.1177/2041247920960973

[CR33] Rusu A, Buta EL (2021) The development of third-generation tetracycline antibiotics and new perspectives. Pharmaceutics 13:2085. 10.3390/PHARMACEUTICS13122085/S1 34959366 10.3390/pharmaceutics13122085 PMC8707899

[CR34] Sagbas S, Butun S, Sahiner N (2012) Modifiable chemically crosslinked poli(κ-carrageenan) particles. Carbohydr Polym 87:2718–2724. 10.1016/J.CARBPOL.2011.11.064

[CR35] Salehi M, Molzemi S (2023) Fabrication and mechanical properties of chitosan/FHA scaffolds. Adv Polym Technol. 10.1155/2023/2758621

[CR36] Sangeetha P, Selvakumari TM, Selvasekarapandian S et al (2020) Preparation and characterization of biopolymer K-carrageenan with MgCl2 and its application to electrochemical devices. Ionics (Kiel) 26:233–244. 10.1007/S11581-019-03193-0/TABLES/6

[CR37] Sultana S, Alzahrani N, Alzahrani R et al (2020) Stability issues and approaches to stabilised nanoparticles based drug delivery system. J Drug Target 28:468–486. 10.1080/1061186X.2020.1722137 31984810 10.1080/1061186X.2020.1722137

[CR38] Sunil L, Prakruthi A, Prashant Kumar PK, Gopala Krishna AG (2020) Coconut Water Nature’s miracle health drink Chemistry, Health Benefits, Packaging, Storage and Technologies : A Review. Indian Coconut Journal 17–25

[CR39] Wu SJ (2012) Degradation of κ-carrageenan by hydrolysis with commercial α-amylase. Carbohydr Polym 89:394–396. 10.1016/J.CARBPOL.2012.03.019 24750735 10.1016/j.carbpol.2012.03.019

[CR40] Zhang G, Meredith TC, Kahne D (2013) On the essentiality of lipopolysaccharide to Gram-negative bacteria. Curr Opin Microbiol 16:779–785. 10.1016/J.MIB.2013.09.007 24148302 10.1016/j.mib.2013.09.007 PMC3974409

[CR41] Zhang S, Peng B, Wang W (2021) Temperature-Responsive hydrogel carrier for reducing adsorption loss of petroleum sulfonates. Langmuir 37:9809–9816. 10.1021/ACS.LANGMUIR.1C01374/ASSET/IMAGES/LARGE/LA1C01374_0011.JPEG 10.1021/acs.langmuir.1c01374 34343430

