# Supplementary material for: Fostering kappa (κ)-carrageenan hydrogels with the power of a natural crosslinker: a comparison between tender coconut water and potassium chloride (KCl) for therapeutic applications
Source: 3 Biotech. 2025 Mar 14;15(4):87. doi: 10.1007/s13205-025-04254-0 (PMC11908996; doi:10.1007/s13205-025-04254-0)
Supplement: Supplementary file 1 — Supplementary file1 (DOCX 524 KB) [file 13205_2025_4254_MOESM1_ESM.docx]

**Fostering** **Κ-carrageenan Hydrogels with the Power of a Natural Crosslinker:**

**A Comparison between Tender Coconut Water and Potassium Chloride for Therapeutic Applications**


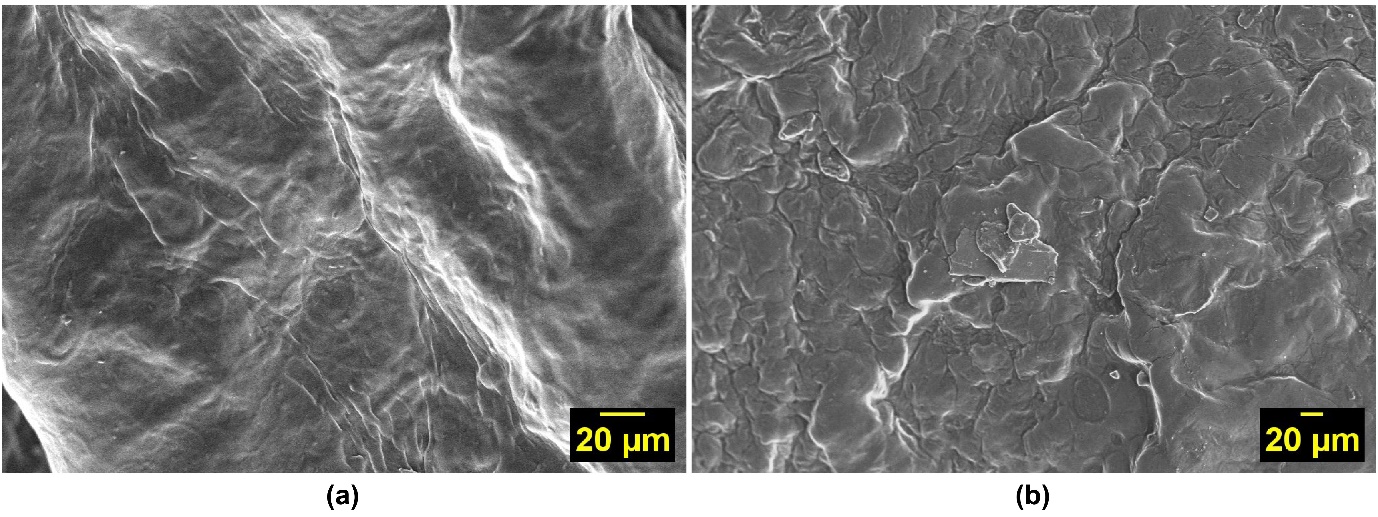


**Figure S1.** (a) SEM micrograph of κ-carrageenan hydrogels (at 1,000×) and (b) SEM micrograph of κ-carrageenan + diclofenac sodium hydrogels (at 500×).


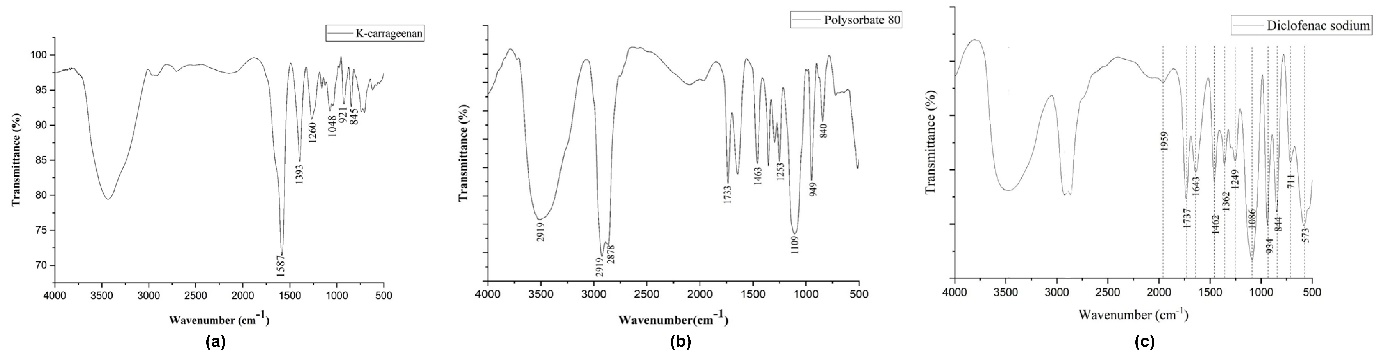


**Figure S2.** FTIR spectrum of (a) κ-carrageenan, (b) polysorbate-80, and (c) diclofenac sodium.
